# Experience of People With Chronic Sinusitis During COVID-19 Lockdown in Saudi Arabia: Insights and Lessons

**DOI:** 10.7759/cureus.40579

**Published:** 2023-06-17

**Authors:** Turki Aldrees, Sharif Almatrafi, Norah Musallam, Ahmad Alroqi

**Affiliations:** 1 Otolaryngology - Head and Neck Surgery, College of Medicine, Prince Sattam Bin Abdulaziz University, Al-Kharj, SAU; 2 Otolaryngology - Head and Neck Surgery, Security Forces Hospital, Riyadh, SAU; 3 Otolaryngology - Head & Neck Surgery, King Saud University, Riyadh, SAU

**Keywords:** covid-19, snot-22, saudi arabia, lockdown, chronic sinusitis

## Abstract

Background

This study aims to evaluate the effect of the COVID-19 lockdown period on chronic rhinosinusitis (CRS) symptoms control.

Methodology

This cross-sectional study was carried out on adult patients (aged ≥18 years) who visited King Abdulaziz University Hospital within six months before the lockdown starting date. Background information, including patients' diagnosis, presence of rhinitis, number of previous surgeries, and medications, was collected. CRS symptom burden was measured using a validated Arabic version of the 22-item Sino-Nasal Outcome Test (SNOT-22). Study participants were required to complete the survey two months after the start of the lockdown.

Results

Out of 66 patients, 43 agreed to participate. The majority of respondents (34, 75.6%) were diagnosed with CRS with nasal polyps. The study revealed no differences between pre- and post-lockdown total symptom scores.

Conclusions

The COVID-19 pandemic has affected the health system worldwide in many ways. Regarding the scope of our study, CRS symptoms, fortunately, did not worsen with the pandemic. This is considered the first reported study to assess such symptom control among people with CRS in Saudi Arabia during the COVID-19 lockdown period.

## Introduction

Chronic rhinosinusitis (CRS) is considered one of the most prevalent chronic conditions, affecting almost 5%-12% of the general population [1]. It is an inflammatory condition represented by inflammation of the paranasal sinuses and lining mucosa for 12 weeks or more [2]. Expectedly, such a chronic disorder requires frequent visits to an otorhinolaryngology clinic and plays a major role in the quality of life (QOL) and health burden worldwide. Diagnosis of CRS can be established clinically in the presence of symptoms such as nasal block, facial pain/pressure, nasal discharge/postnasal drip, and hyposmia/anosmia; through findings of endoscopy such as erythema or edema, presence of discharge, nasal polyp or polypoidal mucosa; and radiologically by scoring the CT images using the Lund-Mackay bilateral scoring system. Treatment options vary between medical, surgical, or both, depending on the severity of the clinical condition and the patient's response [1].

We have lived in the era of a global pandemic - COVID-19 - labeled secondary to SARS-COV-2 [3]. To tackle the pandemic, various strategies, such as quarantines and lockdowns, were implemented worldwide. Before the pandemic, telemedicine was used mainly for palliative care [4]. During COVID-19, healthcare institutes provided good patient care through telemedicine. Regardless of its limitations, telemedicine was used to assess symptom control of CRS patients via phone calls for this study.

This study was conducted during the lockdown period to assess its effects on CRS symptoms control and exacerbation. To the best of our knowledge, no published study in Saudi Arabia has addressed the impact of COVID-19 on people with CRS. This is the first descriptive study that evaluates the effects of the lockdown on people with CRS.

## Materials and methods

This cross-sectional study was conducted between May 27, 2020, and September 15, 2020. Adult patients (aged ≥18 years) with chronic sinusitis who underwent CT scanning and visited King Abdulaziz University Hospital within six months before the lockdown starting date were included. Those who had a recent change in the treatment plan, like newly added medication or a recent (within the last six months) surgical intervention, were excluded. Out of 66 patients, 43 agreed to participate in the study. Background information was collected, including patients' diagnoses, previous surgeries, and medications. CRS symptom burden was measured using a validated Arabic version of the 22-item Sino-Nasal Outcome Test (SNOT-22) [5]. Study participants were required to complete the survey questionnaire two months after the lockdown starting date. SNOT-22 is a validated, 22-item treatment outcome measure applicable to chronic sinusitis. Higher scores on the SNOT-22 survey items suggest lower patient functioning or higher symptom severity (total score range = 0-110). Scoring is done via Likert scale responses where 0 = *No problem*, 1 = *Very mild problem*, 2 = *Mild or slight problem*, 3 = *Moderate problem*, 4 = *Severe problem*, and 5 = *Problem as bad as it can be*. The 22 items of the SNOT-22 survey were recategorized into five distinct domains, namely, rhinological symptoms (Q1, Q2, Q3, Q6, Q21, and Q22), extranasal rhinological symptoms (Q4, Q5, and Q6), ear/facial symptoms (Q2, Q7, Q8, Q9, and Q10), psychological dysfunction (Q14, Q15, Q16, Q17, Q18, Q19, Q20), and sleep dysfunction (Q11, Q12, Q13, Q14, and Q15). CT images were evaluated and staged per the Lund-Mackay bilateral scoring system, where higher scores represent higher bilateral severity of disease (score range = 0-24). All analyses were performed using IBM SPSS Statistics for Windows, Version 25 (IBM Corp., Armonk, NY, USA). The following analyses and calculations were conducted: frequencies, descriptive statistics, and Wilcoxon signed-rank test. The study was approved by the research ethics committee board of Prince Sattam Bin Abdulaziz University, Al-Kharj, Saudi Arabia.

## Results

A total of 43 patients were included in the study, of which 34 (79%) had chronic sinusitis with polyps, four (9%) had chronic sinusitis without nasal polyps, and five (12%) were diagnosed with allergic fungal sinusitis. Among the participants, 35 (81.4%) had previously undergone nasal surgery, while eight (18.6%) had no surgical procedures related to their sinuses.

Differences between pre- and post-lockdown scores

The Wilcoxon signed-rank test was performed to test for pre- and post-lockdown differences. It was chosen due to the non-normality of the data. No significant differences were found in terms of total score (*Z *= -0.58; *P *= 0.564), rhinological symptoms (*Z *= -1.26; *P *= 0.208), extranasal rhinological symptoms (*Z *= -0.06; *P *= 0.950), ear/facial symptoms (*Z *= -0.37; *P *= 0.712), psychological dysfunction (*Z *= -0.16; *P *= 0.872), and sleep dysfunction (*Z *= -0.11; *P *= 0.911) (Table 1).

**Table 1:** Pre-lockdown score mean versus post-lockdown score across total SNOT-22 scores and subdomains. SNOT-22, 22-item Sino-Nasal Outcome Test

Category	Pre-lockdown score, mean (SD)	Post-lockdown score, mean (SD)	*P*-value
Total score	29.71 (25.93)	29.60 (24.71)	0.564
Rhinological symptoms	29.71 (25.93)	11.20 (8.43)	0.208
Extranasal rhinological symptoms	4.58 (3.70)	4.58 (4.30)	0.950
Ear/facial symptoms	5.07 (5.74)	4.98 (5.43)	0.712
Psychological dysfunction	8.96 (10.35)	8.24 (9.49)	0.872
Sleep dysfunction	6.22 (6.84)	6.02 (7.38)	0.911

## Discussion

CRS represents a significant health, social, and economic problem [6]. The significance of CRS is believed to be rising in incidence and prevalence and is comparable to diabetes and heart disease [7]. The primary goal of CRS treatment is to maintain sinonasal symptoms and QOL at acceptable levels for the patient. Therefore, symptom control is one of the principal determinants of the initiation or escalation of CRS treatment [1]. Currently, SNOT-22 is the most commonly utilized and highest-quality sinus-specific QOL instrument available [8].

Regular hospital follow-up visits and medication availability are indispensable for symptom control of chronic medical illnesses. During natural disasters and pandemics, lack of hospital visits and medication supply may adversely affect medication compliance and, consequently, patient symptoms and QOL. Plans must be made in advance to avoid these problems. Studies after Hurricane Charley in Florida suggested that by knowing the prevalence of major chronic conditions, the need for medications should be assessed earlier so that replacement medications may be obtained well in time [9,10].

The World Health Organization (WHO) declared the COVID-19 outbreak, caused by SARS-CoV-2, as a “public health emergency of international concern” on January 31, 2020. The epidemic spread rapidly worldwide within the first two months of the outbreak [11]. In the absence of any pharmaceutical intervention, it was supposed that the only strategy against COVID-19 was to reduce the interaction of susceptible and infectious people through early detection of cases and social distancing [12].

The nationwide lockdown measures in Saudi Arabia were imposed between March 23 and June 20, 2020, to stop the spread of COVID-19 [13]. Lockdowns may worsen symptoms of any chronic disease due to missed follow-up visits. A study by Schlee et al. showed that tinnitus distress increased after the lockdown [14]. Similarly, a study by Davide et al. showed a significant increase in obsession and compulsion severity in patients with obsessive-compulsive disorder after the lockdown [15]. In this context, we hypothesized that lockdown and missed hospital follow-up visits could worsen symptoms and QOL of patients with CRS. Hence, we decided to investigate whether and how the symptoms had changed during the lockdown.

Our study revealed no differences between pre- and post-lockdown total symptom scores. A possible explanation is that the concern of patients about disease recurrence drove them to comply more with the available medical treatment. Another factor that might have contributed to this result is the free consultation and medication delivery service implemented by the Saudi Ministry of Health during the lockdown, using its preexisting online application *Seha* to refill and deliver medications to patients throughout the kingdom [16].

A study by Tait et al. proved that Pulmicort (Pulmican Respules®, Kuhnil Inc., Seoul, Korea) irrigation resulted in a clinically meaningful improvement in self-reported functional status and QOL measures as well as objective measures of CRS [17]. Surprisingly, in our study, the pre-lockdown total scores for Pulmicort irrigation users improved after the lockdown, but their rhinological symptoms scores worsened. However, other factors that were not addressed, such as medication compliance and whether participants followed the correct way of administration, may have played a role in these results.

A study by Senior et al. reported improved subjective results following functional endoscopic sinus surgeries. This improvement can be maintained in the long term with appropriate postoperative management. They also found that prior surgery can affect surgery outcomes [18]. This study showed that those who underwent two surgeries had more pre-lockdown total scores than post-lockdown total scores. However, it is still unclear if this is due to the more chronic and severe spectrum of the disease or if other variables within the surgeon’s control may allow for improved outcomes.

Patients diagnosed with allergic fungal sinusitis (AFS) had higher pre-lockdown than post-lockdown rhinological symptoms. However, only five (11%) participants were diagnosed with AFS, and other factors like seasonality and allergen exposure may have played a role.

Bhattacharyya et al. evaluated the relationship of paranasal sinus symptoms with coronal CT findings. They found that patient-based reports of paranasal sinus symptoms failed to correlate with findings on CT scans [19]. In this study, the preoperative Lund-Mackay bilateral scoring system was used to score the CT scans, and it was not significantly correlated with the pre-lockdown SNOT-22 scores.

A study by Alkhamees et al. showed that nearly one-fourth of the sampled general population in Saudi Arabia experienced moderate-to-severe psychological impact due to the COVID-19 pandemic [20]. Moreover, high levels of anxiety and depression are typical in patients with CRS, and psychiatric comorbidities are associated with increased CRS symptoms [21]. The present study had no statistically significant difference in the overall psychological scores before and after the lockdown. However, self-reporting of the psychological impact may not always be aligned with objective assessment by mental health professionals.

There are certain limitations to the study. First, the cross-sectional study design provides only a snapshot of responses at a particular point in time. However, a longitudinal study was not feasible with the prevalent social distancing and lockdown situation at that time. Second, it did not cover all the potential factors that may influence participants’ symptoms because the time frame was too short to evaluate them. Despite the aforementioned limitations, this study is the first descriptive study to compare the effects of the lockdown on CRS patients and can be preliminary research to build on.

## Conclusions

The COVID-19 pandemic has conclusively affected health systems worldwide in many ways. Regarding the scope of our study, as stated previously, CRS symptoms, fortunately, did not worsen with the pandemic lockdown. The free online consultations and medication refill delivery services might have contributed to this. However, further longitudinal studies are required to address the effect of other factors that may moderate the responses of patients.
